# CD45 Isoform Profile Identifies Natural Killer (NK) Subsets with Differential Activity

**DOI:** 10.1371/journal.pone.0150434

**Published:** 2016-04-21

**Authors:** Ewelina Krzywinska, Amelie Cornillon, Nerea Allende-Vega, Dang-Nghiem Vo, Celine Rene, Zhao-Yang Lu, Christine Pasero, Daniel Olive, Nathalie Fegueux, Patrick Ceballos, Yosr Hicheri, Michal Sobecki, Jean-François Rossi, Guillaume Cartron, Martin Villalba

**Affiliations:** 1 INSERM U1183, Université de Montpellier 1, UFR Médecine, Montpellier, France; 2 Institute for Regenerative Medicine and Biotherapy (IRMB), CHU Montpellier, Montpellier, 34295, France; 3 Centre de Cancérologie de Marseille, Plateforme d'Immunomonitoring en Cancérologie, INSERM, U1068, Institut Paoli-Calmettes, Aix-Marseille Université, UM 105, CNRS, UMR7258, Marseille, France; 4 Département d'Hématologie Clinique, CHU Montpellier, Université Montpellier I, 80 avenue Augustin Fliche, 34295, Montpellier, France; 5 Institute for Integrative Biology of the Cell (I2BC), Genome Biology Department CNRS - UMR9198, Gif-sur-Yvette, France; 6 CNRS UMR5235, Université de Montpellier, Montpellier, France; INSERM-Université Paris-Sud, FRANCE

## Abstract

The leucocyte-specific phosphatase CD45 is present in two main isoforms: the large CD45RA and the short CD45RO. We have recently shown that distinctive expression of these isoforms distinguishes natural killer (NK) populations. For example, co-expression of both isoforms identifies *in vivo* the anti tumor NK cells in hematological cancer patients. Here we show that low CD45 expression associates with less mature, CD56^bright^, NK cells. Most NK cells in healthy human donors are CD45RA^+^CD45RO^-^. The CD45RA^-^RO^+^ phenotype, CD45RO cells, is extremely uncommon in B or NK cells, in contrast to T cells. However, healthy donors possess CD45RA^dim^RO^-^ (CD45RA^dim^ cells), which show immature markers and are largely expanded in hematopoietic stem cell transplant patients. Blood borne cancer patients also have more CD45RA^dim^ cells that carry several features of immature NK cells. However, and in opposition to their association to NK cell progenitors, they do not proliferate and show low expression of the transferrin receptor protein 1/CD71, suggesting low metabolic activity. Moreover, CD45RA^dim^ cells properly respond to *in vitro* encounter with target cells by degranulating or gaining CD69 expression. In summary, they are quiescent NK cells, with low metabolic status that can, however, respond after encounter with target cells.

## Introduction

NK cells recognize and eliminate blood-borne cancer cells. However, these tumor cells use different mechanisms for immune escape [1], i.e. inducing NK cell dysfunction [2]. Therefore a significant number of patients with hematological malignancies show limited long-term survival. Some treatment options include new chemicals that can be associated with immunotherapy i. e. cell therapy to boost the immune response [3, 4]. In this context, clinical-grade production of allogeneic NK cells is efficient [5] and NK cell–mediated therapy after hematopoietic stem cell transplantation (HSCT) seems safe [6–8]. However, NK cells are not a homogenous population and different subsets have different physiological activities. Moreover, different *in vitro* protocols for NK cell expansion and activation (*e*.*g*., cytokines *vs* targets cells) give rise to different immunophenotypes [9]. In this context, efficient expansion and/or activation protocols produce cells able to overcome all tested anti apoptotic mechanisms developed by tumor cells [10]. The presence of other immune cell types, which favor effective NK cell activation through the production of cytokines such as interferon-α (IFN-α) or interleukin-15 (IL-15), probably mediates optimal NK cell expansion [11] [12].

In peripheral blood, human NK cells are mostly CD3^-^CD56^dim^ cells with high cytotoxic activity, while CD3^-^CD56^brigth^ cells excel in cytokine production [13]. *In vitro* evidence indicates that CD56^bright^ NK cells are precursors of CD56^dim^ NK cells and this might also be the case *in vivo* [14]. In addition, combined analysis of CD56 and CD16 expression during NK cell development indicates that their profiles changes as follows: CD56^brigth^CD16^-^ → CD56^brigth^CD16^dim^→ CD56^dim^CD16^dim^→ CD56^dim^CD16^+^. Additional markers can be used to identify specific subsets within these NK cell populations [15, 16]. Due to the clinical interest of NK cells, it is therefore highly relevant to precisely identify NK cell populations with specific or pronounced functions.

We have recently shown that expression of different CD45 isoforms in human NK cells identify separate populations [17]. CD45 is a protein tyrosine phosphatase that is specifically expressed in leucocytes [18]. CD45 regulates receptor signaling by direct interaction with components of the receptor complexes or by dephosphorylating and activating various Src family kinases (SFK) [19]. CD45 activity is critical for efficient immune response, because its deficiency results in severe combined immunodeficiency (SCID) in mice [20–22] and humans [23, 24].

In T cells, CD45 can be expressed as one of several isoforms by alternative splicing. The largest isoform CD45RA is expressed on naïve T cells. Activated and memory T lymphocytes express the shortest CD45 isoform, CD45RO, which lacks RA, RB, and RC exons. This shortest isoform facilitates T cell activation. CD45 variants with short extracellular domain homodimerize more easily than those with large extracellular domain [25]. The activity of CD45 is regulated by dimerization, which inhibits the activity of CD45. The extent of dimerization and inhibition is inversely proportional to the size of the extracellular domain: larger CD45 isoforms such as CD45RA dimerize less efficiently [19].

NK cells expressing the long, CD45RA, and the short, CD45RO, isoforms in the same NK cells show higher antitumor activity in hematological cancer patients [17]. Here we describe that expression of CD45 isoforms give NK cells different functional properties. Specifically, decreased expression of CD45RA, CD45RA^dim^ cells, is observed in immature cells that still do not proliferate or show high metabolism. Nevertheless, they show cytotoxic activity and CD69 expression after *in vitro* encounter with target cells. Hence, we identify a new population of quiescent, immature, NK cells that recognizes target cells.

## Materials and Methods

### 2.1. Cell culture

The K562 cell line (ATCC CCL 243) and the lymphoblastoid EBV cell line PLH (IHW Number: 9047) were maintained in logarithmic growth in RPMI 1640 medium (Gibco^®^ GlutaMAX^™^ media) with 10% fetal bovine serum (FBS) (Gibco^®^). Cells were cultured at 37°C in a humidified chamber with 5% CO_2_ in air, and passaged 1:10 twice a week.

### 2.2. Peripheral blood mononuclear cell (PBMC) purification

Bone marrow and peripheral blood samples were obtained from patients with different hematological diseases and from healthy donors after informed consent. Cells were purified by Ficoll-Hypaque (Sigma) density-gradient centrifugation. Briefly, 3–6 ml of 1:2 diluted blood or 1:3 diluted bone marrow samples in RPMI were added on top of 5 ml of Histopaque. Cells were centrifuged at 1600 rpm and at 20°C without break for 30 minutes. Mononuclear cells were collected from the interlayer white ring. After washing in RPMI, cells were suspended in complete RPMI medium supplemented with 10% FBS (Invitrogen).

### 2.3. *In vitro* NK cell stimulation protocol

PBMCs, 1.10^6^ cells/ml, were stimulated during 10 or 20 days with a high dose of IL-2 (1000 U/ml, eBiosciences) or with the lymphoblastoid EBV cell line PLH together with IL-2 (100 U/ml) and IL-15 (5 ng/ml, Miltenyi).

### 2.4. Selection of patients

Data and samples from patients with different hematological cancers were collected at the Clinical Hematology Department of the CHU Montpellier, France, after patient’s written consent that were collected and kept by the CHU Montpellier [17, 26]. Following French regulations, patients were enrolled in two independent clinical programs approved by the “Comités de Protection des Personnes Sud Méditerranée I”: ref 1324 and HEMODIAG_2020 (ID-RCB: 2011-A00924-37). All samples from cancer patients were collected at diagnosis and included **HD,** Healthy donor (n_bs_ = 10); **MM**, multiple myeloma (n_bs_ = 19, n_bms_ = 20); **B-CLL**, B-cell chronic lymphocytic leukemia (n_bs_ = 15); **BCL**, B-cell lymphoma (n_bs_ = 14); **AML**, acute myeloid leukemia (n_bs_ = 14); **bs**, blood samples; **bms**, bone marrow samples.

### 2.5. Multicolor Staining of Cell Surface Markers

PBMCs were stained with 7AAD (Beckman) to identify viable cells and with the following anti-CD25-FITC, -CD45RO-FITC, -CD161-FITC, -CD3-PE, -CD19-PE, -CD62L-PE, -CD69-PE, -CD138-PE, -CD314(NKG2D)-PE, -CD3-ECD, -CD19-ECD, -CD38-ECD, -CD56-PECy7, CD3-APC, -CD56-APC, -GzB-AlexaFluor700, -CD19-AlexaFluor700, -CD20-APC-AlexaFluor750, -CD45-APC-AlexaFluor750, -CD45RA-APC-AlexaFluor750, -CD5-PacificBlue, -CD16-PacificBlue, -CD57-PacificBlue, -CD45-KromeOrange, -CD16-KromeOrange (Beckman), -CD158b-FITC, -CD158a-PE, -CD107a-HV500, -Ki-67-V450 (BD Biosciences), -CD45RA-FITC, -CD45RO-PE, -CD159a(NKG2A)-PE, -CD335(NKp46)-PE, -CD94-PE-Vio770, -CD335(NKp46)-PE-Vio770, -CD45RO-APC, -CD14-VioBlue, -CD19-VioBlue, -CD158e-VioBlue (Miltenyi Biotec) and -CD71-APC (ImmunoTools) antibodies against surface markers for cell phenotyping. Briefly, 1x10^6^ cells were incubated with the different antibodies in PBS/2% FBS at 37°C for 30 minutes. Cells were then washed and suspended in 200–250 μl PBS/2% FBS and staining was analyzed using a Gallios flow cytometer (Beckman) and the Kaluza software.

Viable lymphocytes were gated using FSC/SSC and 7AAD staining. B lymphocytes (CD19+), T lymphocytes (CD3+CD56-) and NK cells (CD56+CD3-) were differentiated based on CD19, CD3 or CD56 expression. NK cells were then separated in four distinct populations based on CD45RA and CD45RO expression: CD45RA^+^RO^-^ (**CD45RA**), CD45RA^+^RO^+^ (**CD45RARO**), CD45RA^dim^RO^-^ (**CD45RA**^**dim**^), CD45RA^dim^RO^+^ (**CD45RA**^**dim**^**RO**). These different populations were then analyzed for CD16, CD57, CD62L, CD69, CD71, CD94, CD107a, CD158a, CD158b, CD158e, CD159a (NKG2A), CD161, CD314 (NKG2D), CD335 (NKp46), Ki-67, GzmB expression and cell size/granularity (FSC/SSC).

### 2.6. *In vitro* CD107a Degranulation Assay

After PBMC purification and NK cell quantification, 3 million cells were incubated at 37°C for 4h or overnight with K562 target cells at an Effector (NK cell): Target ratio of 1:10 in a final volume of 500μl (RPMI Glutamax with 10% FBS and 10u/ml IL2). The medium also contained 1.5μl anti-CD107a antibody (BD Biosciences, Franklin Lakes, NJ) and 1μl monensin to prevent CD107a degradation (BD Golgi-Stop BD Biosciences). Then, cells were resuspended in 50μl of an antibody cocktail containing 7AAD, the anti-CD45RO-FITC, -CD69-PE, -CD19-ECD, -CD56-PECy7, -CD3-APC, -CD45RA-APCAlexaFluor750, -CD107a-HV500 and -CD16-KO antibodies (BD Biosciences, Beckman). Samples were analyzed on a Beckman Coulter FACS Gallios flow cytometer using the Kaluza software. Events were initially gated on forward and side scatter (SSC) to identify lymphocytes. A bivariate plot of CD56 versus CD3 was used to acquire at least 10,000 NK cells.

### 2.7. Multicolor Staining for Cell Surface and Intracellular Markers

After PBMC purification, 1 million cells were pre-blocked by incubation with 10% normal human serum at RT for 15 min and then stained with 50μl of the PANEL Ki-67 antibody cocktail against cell surface markers (anti-CD45RO-PE, -CD19-ECD, -CD56-PC7, -CD3-APC, -CD45RA-APCAlexaFluor750 and -CD16-KO antibodies) (BD Biosciences, Beckman). Cells were washed twice with Staining Buffer and resuspended in 250μl BD Cytofix/Cytoperm solution at 4°C for 20 min. Cells were washed twice in BD Perm/Wash solution. Next, cells were fixed/permeabilized in 50μl BD Perm/Wash solution containing an antibody cocktail against intracellular markers (anti-GzB-AlexaFluor700, -Ki-67-V450) as described in the figures at 4°C for 30 minutes in the dark. Cells were washed twice in BD Perm/Wash solution and resuspended in Staining Buffer prior to flow cytometric analysis on a Beckman Coulter FACS Gallios flow cytometer using the Kaluza software. Events were initially gated on forward and side scatter (SSC) to identify lymphocytes. A bivariate plot of CD56 versus CD3 was used to acquire at least 10,000 NK cells.

### 2.8. Statistics

All the experiments shown in the figures were performed at least with samples from six patients for each malignancy and the same number of healthy donors (HD). The statistical analysis was performed using the Student t test: *p<0.05; **p<0.01; ***p<0.001. Average values were expressed as mean plus or minus the standard error (SD).

## Results

### 3.1. CD45 is a marker of mature human NK cells

**Fig 1:**
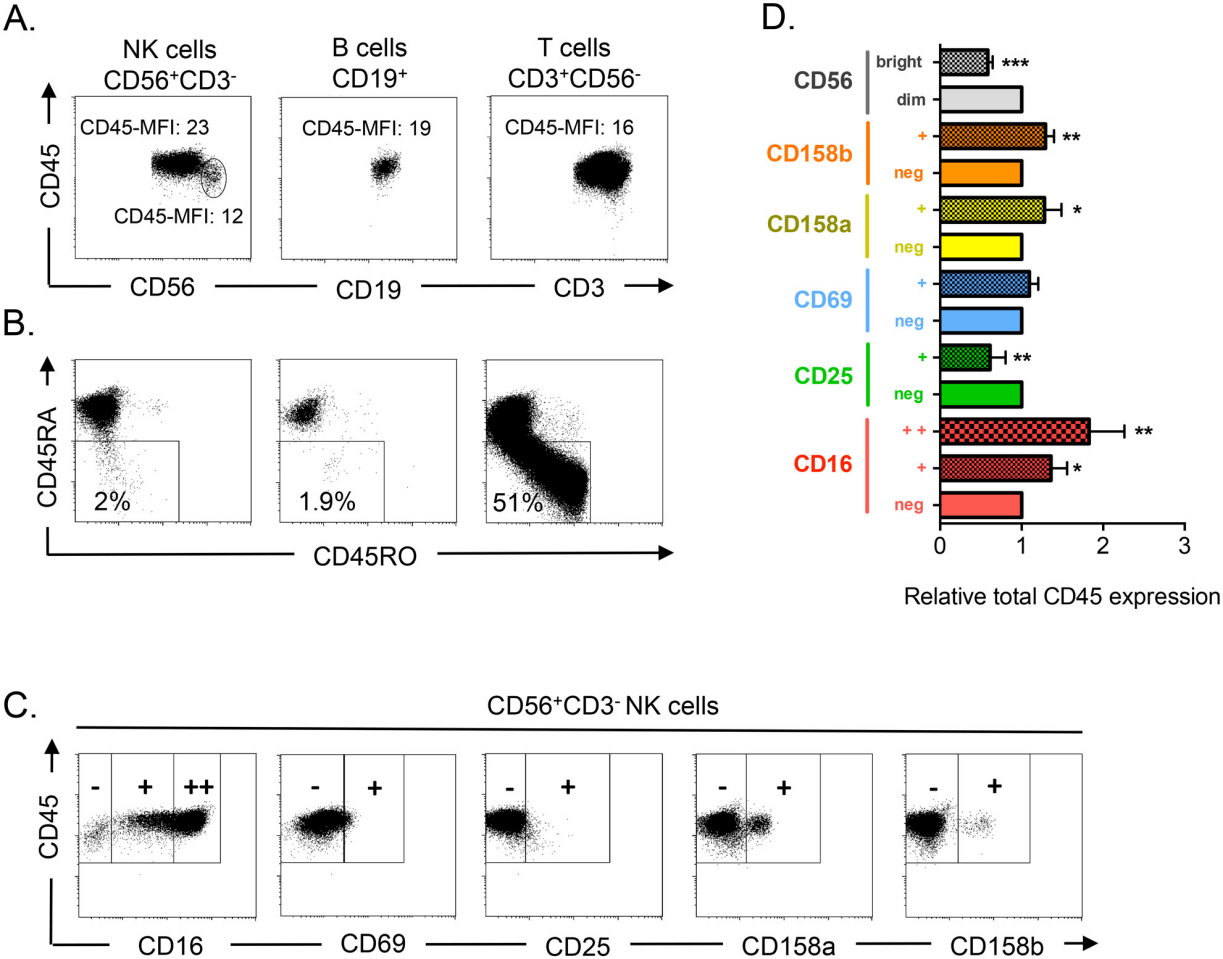
CD45 expression correlates with markers of NK cell maturation. A) After purification, PBMCs from healthy donors were stained for FACS analysis with anti-CD19 (B cells, CD19^+^), -CD3 (T cells, CD3^+^CD56^-^) and -CD56 (NK cells, CD56^+^CD3^-^), to identify the different lymphocyte populations, and also with anti-CD45. CD45 mean fluorescence intensities (MFI) are given for each population and for CD56^bright^ NK cells. B) PBMCs were stained as in (A) and with anti-CD45RA and -CD45RO antibodies. The percentage of CD45RA^dim^ cells is given for each lymphocyte population. C) Cells were stained as in (A) and with antibodies against the NK cell markers CD16, CD25, CD69, CD158a and CD158b to assess their co-expression with CD45 in NK cells. D) The bars show the mean ± SD (n = 4) of the relative CD45 expression in different NK cell populations. To avoid inter-donor variations, the histogram shows the relative expression of CD45 in each NK cell subset, which was defined based on the positive or negative expression of a given marker. For comparison, CD45 expression in the populations that does not, or barely, expresses that marker was arbitrarily set to 1.

To determine whether high CD45 expression is a marker of cell maturation also in human NK cells as in other lymphocyte types [27], we analyzed CD45 expression in the three main types of human lymphocytes (T, B and NK cells) derived from PBMCs (Fig 1A). B and T cells showed relatively homogenous populations with comparable levels of CD45 expression. CD56^bright^ (thus relatively immature) NK cells showed lower total CD45 expression, in agreement with the notion that CD45 is a marker of lymphocyte maturation. Most NK and B cells expressed CD45RA, whereas very few expressed CD45RO (Fig 1B). In contrast, a large T cell population expressed CD45RO instead of CD45RA.

We then analyzed the association of total CD45 expression with known NK cell markers (Fig 1C and 1D). Higher CD45 expression in NK cells was associated with expression of the KIRs CD158a and CD158b and of CD16 (markers of NK cell maturation [15, 28]). Conversely, CD45 expression was lower in cells positive for CD25 (a marker of cell proliferation). CD69-positive cells, a marker associated with NK cell cytolytic activity [29], also expressed higher CD45 levels than CD69-negative cells, although this difference was not statistically significant. These results indicate that in NK cells, total CD45 expression is mainly associated with high expression of NK cell maturation markers, such as CD16 and KIRs, and with low expression of CD56 and CD25 (markers of immature NK cells).

### 3.2. Expression of different CD45 isoforms *in vivo*: healthy donors (HD)

**Fig 2:**
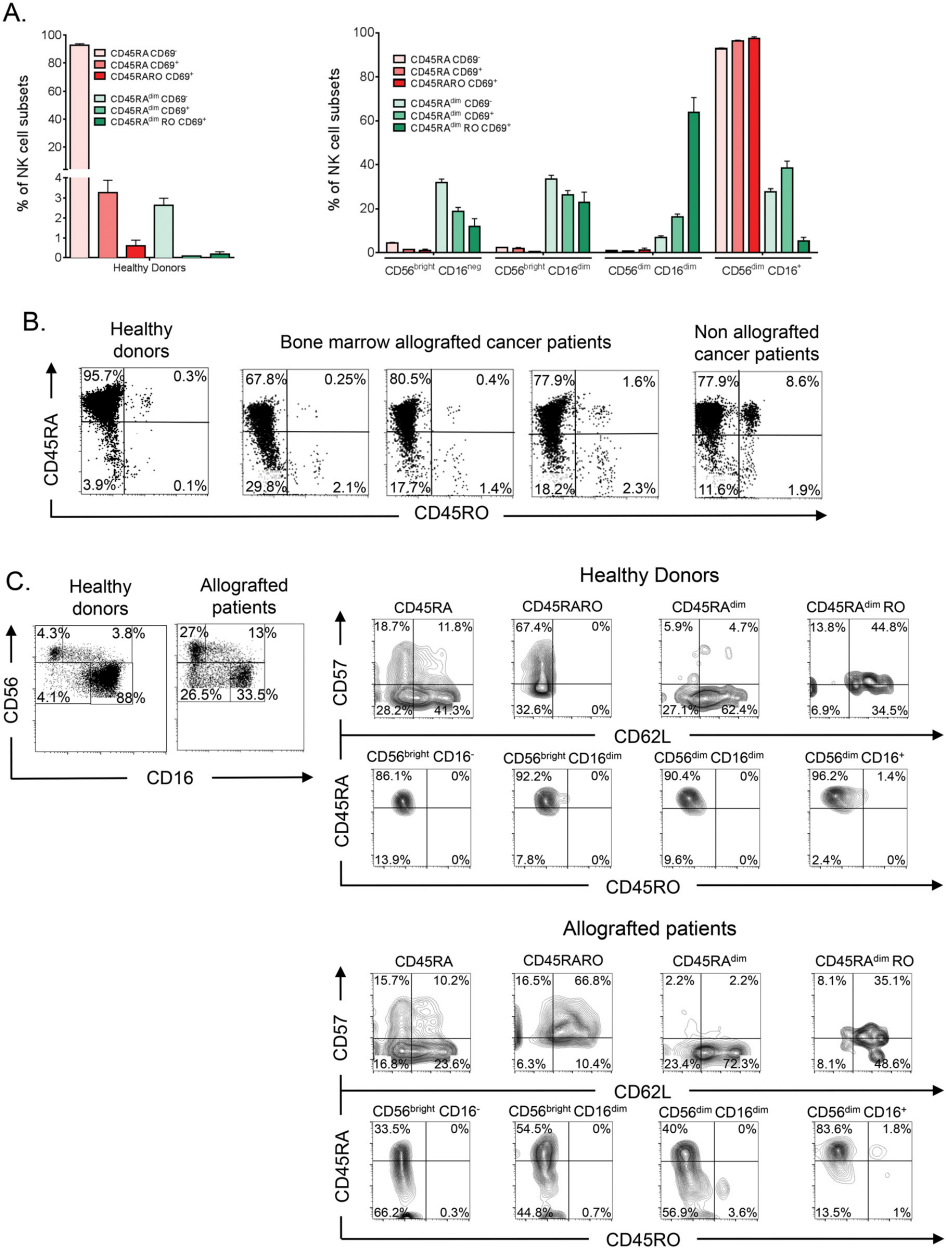
NK cell subsets in cancer patients allografted with HSC. A) After purification, PBMCs from healthy donors were stained as in Fig 1 and also with anti-CD16, to identify NK cell subsets at different stage of maturation, and with -CD45RA, -CD45RO and -CD69 antibodies. The bars in the left panel show the percentage (mean ± SD) of the six NK cell subsets based on their CD45 and CD69 expression profile. The right panel depicts the abundance of these six populations at four NK cell maturation stages, based on CD56 and CD16 expression. B) PBMCs from healthy donors, allografted patients with hematological cancer and one non-allografted patient with multiple myeloma were stained as in (A) and CD45RA and CD45RO expression analyzed. C) Samples in (B) were also stained with anti- CD62L and -CD57 antibodies.

Hence, few NK cells express CD45RO compared to T cells. To better understand the role of the different CD45 populations, we performed a more detailed analysis of cell surface marker expression in NK cells from healthy donors. We divide NK cells in different subsets regarding CD45RA, CD45RO and CD69 expression. CD45RO cells almost always expressed CD69 [17], and hence CD45ROCD69^-^ were not present. We showed that the vast majority of NK cells in HD were CD45RA^+^ and RO^-^ (CD45RA) and CD69^-^, only 4% of them expressed CD69 (Fig 2A, left panel and 2B). Among the 3% of NK cells with low CD45RA expression (CD45RA^dim^), we found very few CD69^+^ cells. Furthermore, based on the expression of CD56 and CD16 (Fig 2A, right panel), CD45RA cells could be mainly considered fully mature cells, whereas CD45RA^dim^RO^-^ (CD45RA^dim^) cells were consistently found in immature populations. The very few CD45RA^+^RO^+^ (CD45RARO) cells were CD56^dim^CD16^+^, whereas CD45RA^dim^RO^+^ (CD45RA^dim^RO) cells were principally CD56^dim^CD16^dim^ (Fig 2A and 2C). These results suggest that CD45RA and CD45RO expression are partly associated with NK cell maturation in healthy donors.

### 3.3. Expression of different CD45 isoforms *in vivo*: allografted patients

To investigate the relationship between the expression of CD45 isoforms and NK cell development, we investigated the NK cell profiles in HSCT patients 3 months after transplantation. We chose these patients because it was expected that they possessed an immature immune system that is in process of development. We confirmed it in blood samples where the number of immature, based on CD56 and CD16 expression, NK cells was higher than in healthy controls (Fig 2C, left panels). These CD56^bright^ cells were CD16^-^, suggesting that they were *de novo* produced NK cells and not CD56^dim^ cells that gained CD56 expression following activation. Interestingly, allografted patients showed more CD45RA^dim^ cells than HD (Fig 2B). These cells, as in HD, are mainly present in the most immature compartments regarding CD56/CD16 expression (Fig 2C).

During *in vivo* maturation CD56^bright^ cells become CD56^dim^CD62L^+^CD57^-^ cells that produce perforin, while maintaining high IFN-γ production in response to cytokines [13, 30]. Then, CD56^dim^CD62L^-^CD57^+^ cells show low response to cytokines and higher cytotoxic capacity [13, 31]. In HD CD45RO expression is more associated to CD57 than to CD62L (Fig 2C and [17]). In patients there was variability between donors with an increase in CD62L expression in all subsets (Fig 2C and S1 Fig). It was difficult in this context to give a “grade” of maturity to the CD45 populations due to the global increase in CD62L.

The previously described patients were grafted with bone marrow as a treatment for different hematological cancers. These patients, in addition to higher CD45RA^dim^ NK cell numbers, present a larger CD45RO^+^ population than HD (Fig 2B; [17]). Hence, the presence of CD45RO^+^ cells in allografted patients was probably reminiscent to the previous or current presence of hematopoietic tumor cells [17].

**Fig 3:**
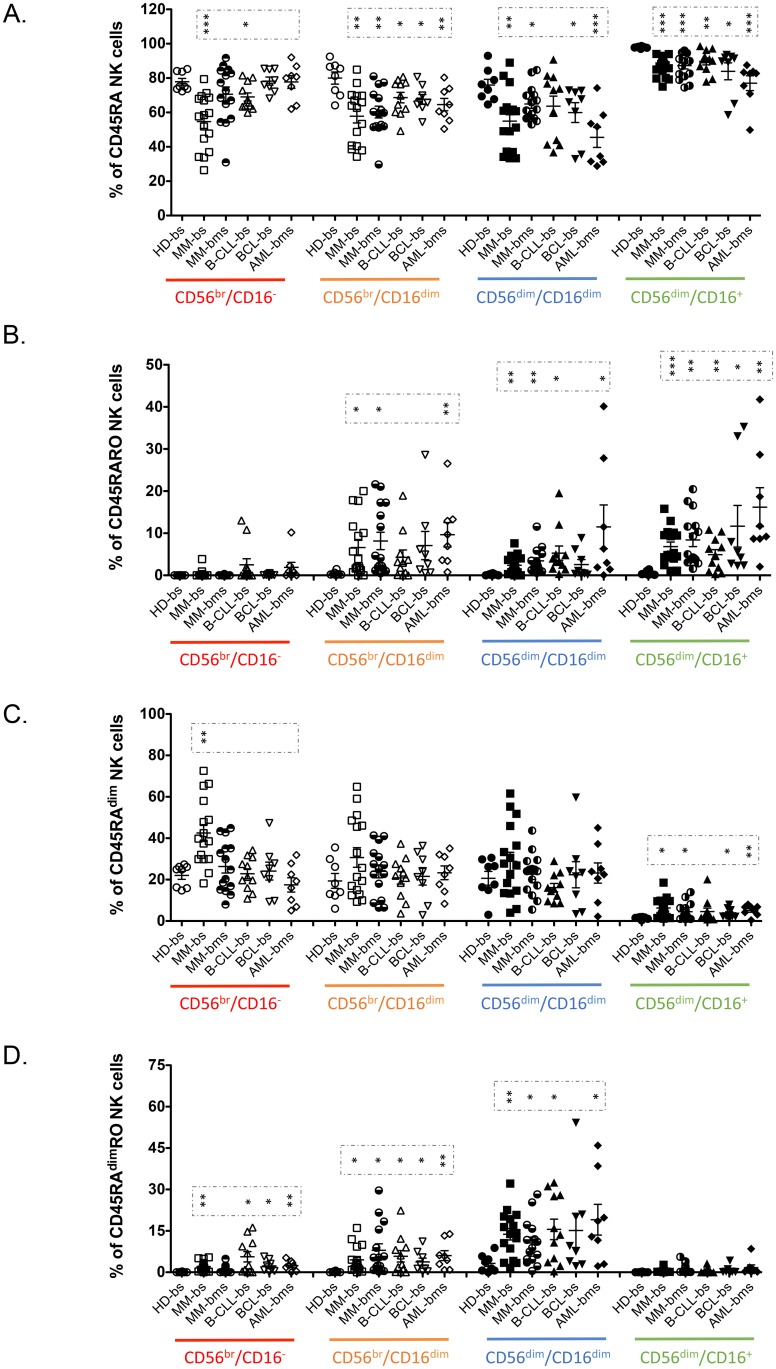
Patients with hematological malignancies have specific NK cell subset profiles that correlate with maturation status. PBMCs from blood samples (bs) of healthy donors and of patient with different hematological cancers or from bone marrow (bms) of the patient with MM or AML were stained as in Fig 2. The percentage of CD45RA, CD45RARO, CD45RA^dim^ and CD45RA^dim^RO cells at the different stage of NK maturation (CD56/16 expression) was depicted in the graphic. Each point represents a donor. The mean ± SD and the statistical analysis are also shown. **HD,** Healthy donor; **MM**, multiple myeloma; **B-CLL**, B-cell chronic lymphocytic leukemia; **BCL**, B-cell lymphoma; **AML**, acute myeloid leukemia; **bs**, blood samples; **bms**, bone marrow samples.

We next confirmed the increase on the CD45RA^dim^, CD45RARO and CD45RA^dim^RO in these patients (Fig 3) and investigated CD57 and CD62L expression in our cohort of multiple myeloma (MM) patients (S2 Fig). This analysis confirmed the tendency observed in allografted patients: CD45RO^+^ cells expressed higher numbers of CD57^+^ cells, but not of CD62L^+^ cells (S2 Fig). CD45RA^dim^ cells expressed lower CD57 levels but not CD62L. These data once more supported a role for the expression of different CD45 isoforms in NK cell maturation.

### 3.4. Relationship between CD45 isoforms and CD69 expression

**Fig 4:**
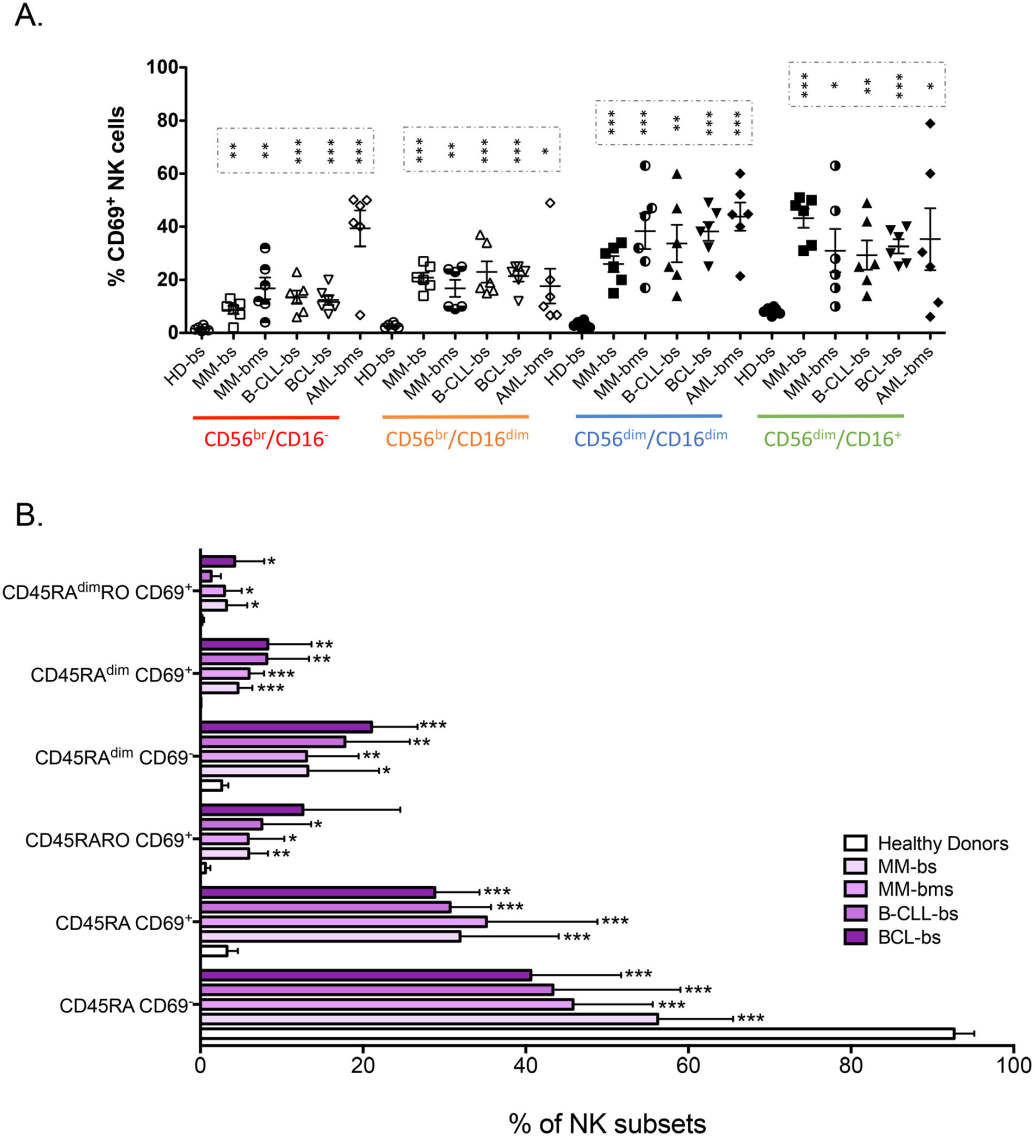
CD69, CD45RA and CD45RO identify different NK cell subsets. A) PBMCs from healthy donors (HD) and patients with different hematological malignancies were purified as in Fig 1 and the percentage of CD69^+^ cells at different stages of NK cell maturation (CD56/CD16) was calculated. The mean ± SD is also depicted. B) Percentage of NK cells in the different NK cell populations based CD45RA, CD45RO and CD69 expressions in PBMCs from healthy donors (HD) and patients with different hematological malignancies. Bars represent the mean ± SD for each medical condition.

CD69 expression increases after NK cell stimulation and is considered a *bona fide* marker of NK cell activation [32], including *ex vivo* [33]. In HD, CD69 was basically expressed by fully mature CD56^dim^CD16^+^ cells (Fig 4A), whereas in patients with hematological malignancies CD69^+^ cells were detected in all CD56/CD16 NK cell subsets, independently of their maturation (Fig 4A). Analysis of CD69 expression in the different CD45 populations in patients showed the presence of larger amounts of CD45RA and CD45RA^dim^ cells that co-expressed also CD69 and CD45RO compared to healthy controls (Fig 4B).

In healthy donors, CD45RA^dim^ cells were mainly CD69^-^ (Fig 4B). CD45RA^dim^ cells were significantly increased in patients and many were also CD69^+^. However, reduction of CD45RA expression was not associated with gain of CD69 expression. In fact, patients’ samples were more enriched in CD45RA^dim^ CD69^-^ than in CD45RA^dim^ CD69^+^ cells.

In conclusion, CD69^+^ cells were found in all maturation subsets in patients (Fig 4A) whereas CD45RA^dim^ cells were mostly in immature populations (CD56^bright^ and CD56^dim^CD16^dim^) (Fig 3). This suggests that loss of CD45RA and gain of CD69 expression identify two different physiological processes and that these two populations might have different functions.

### 3.5. Expression of different CD45 isoforms and CD69 identifies different NK cell functions *ex vivo*

**Fig 5:**
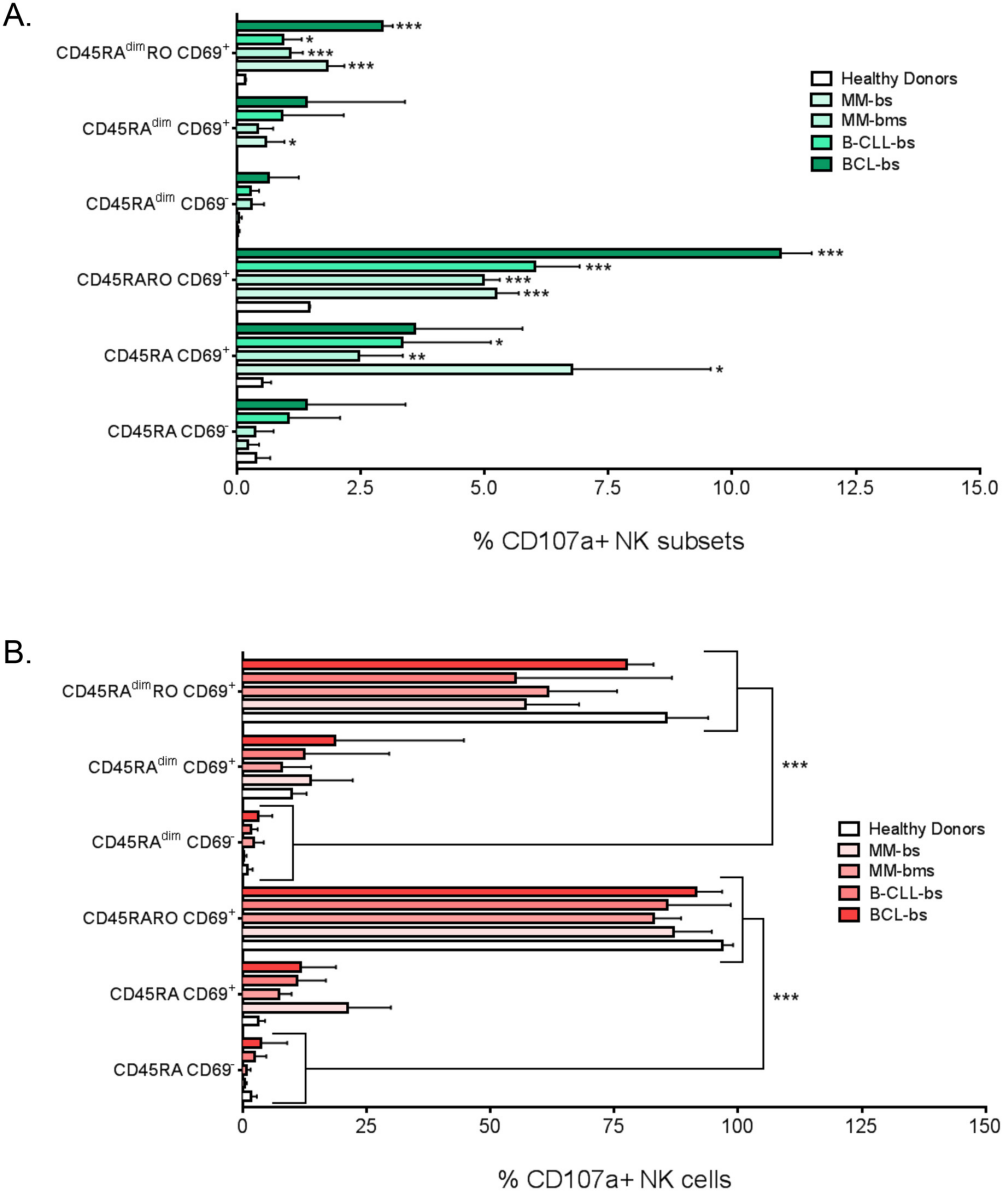
CD45RO identifies degranulating NK cells. PBMCs from healthy donors (HD) and patients with different hematological malignancies were purified as in Fig 1. A) Percentage of the total NK cells that were CD107a^+^ and expressed the corresponding markers to be considered in one of the six NK cell subsets based on CD45RA/CD45RO/CD69 expression. B) Percentage of NK cells in the different subsets that were CD107a^+^. Bars represent the mean ± SD for each medical condition. To identify the function of the different NK cell subsets, we firstly assessed cell degranulation by *ex vivo* staining of PBMCs with anti-CD107a antibodies. Fig 5A depicted the % of CD107a^+^ NK cells and in which NK subset they were regarding expression of CD5RARO and CD69. For example in B-CLL there were 21% of CD107a^+^ NK cells; of those, 12% were CD5RARO CD69^+^, 3% CD45RA CD69^+^, 3% CD45RA^dim^RO CD69^+^, etc. Hematological cancer patients showed a large increase in CD107a^+^ cells. Most of these cells were CD69^+^. Fig 5B showed the % of NK cells in each of the 6 populations that are CD107a^+^, which was basically 100% for CD5RARO CD69^+^ cells. CD45RA^dim^RO CD69^+^ cells were also basically CD107a^+^. Overall, CD107a expression associated with CD45RO expression and was more related to CD69^+^ than to CD69^-^ cells and CD45RA down-regulation did not affect CD107a expression.

To identify the function of the different NK cell subsets, we firstly assessed cell degranulation by *ex vivo* staining of PBMCs with anti-CD107a antibodies. Fig 5A depicted the % of CD107a^+^ NK cells and in which NK subset they were regarding expression of CD45RA, CD45RO and CD69. Hematological cancer patients showed a large increase in CD107a^+^ cells. Most of these cells were CD69^+^. For example in BCL there were 21% of CD107a^+^ NK cells; of those, 11.5% were CD5RARO CD69^+^, 3% CD45RA CD69^+^, 3% CD45RA^dim^RO CD69^+^, 1.5% CD45RA CD69^-^, 1.5% CD45RA^dim^RO CD69^-^ and 0.5% CD45RA^dim^ CD69^-^. Fig 5B showed the % of NK cells in each of the 6 populations that are CD107a^+^, which was basically 100% for CD5RARO CD69^+^ cells. Most CD45RA^dim^RO CD69^+^ cells also expressed CD107a^+^. Overall, CD107a expression associated with CD45RO expression and was more related to CD69^+^ than to CD69^-^ cells and CD45RA down-regulation did not affect CD107a expression.

**Fig 6:**
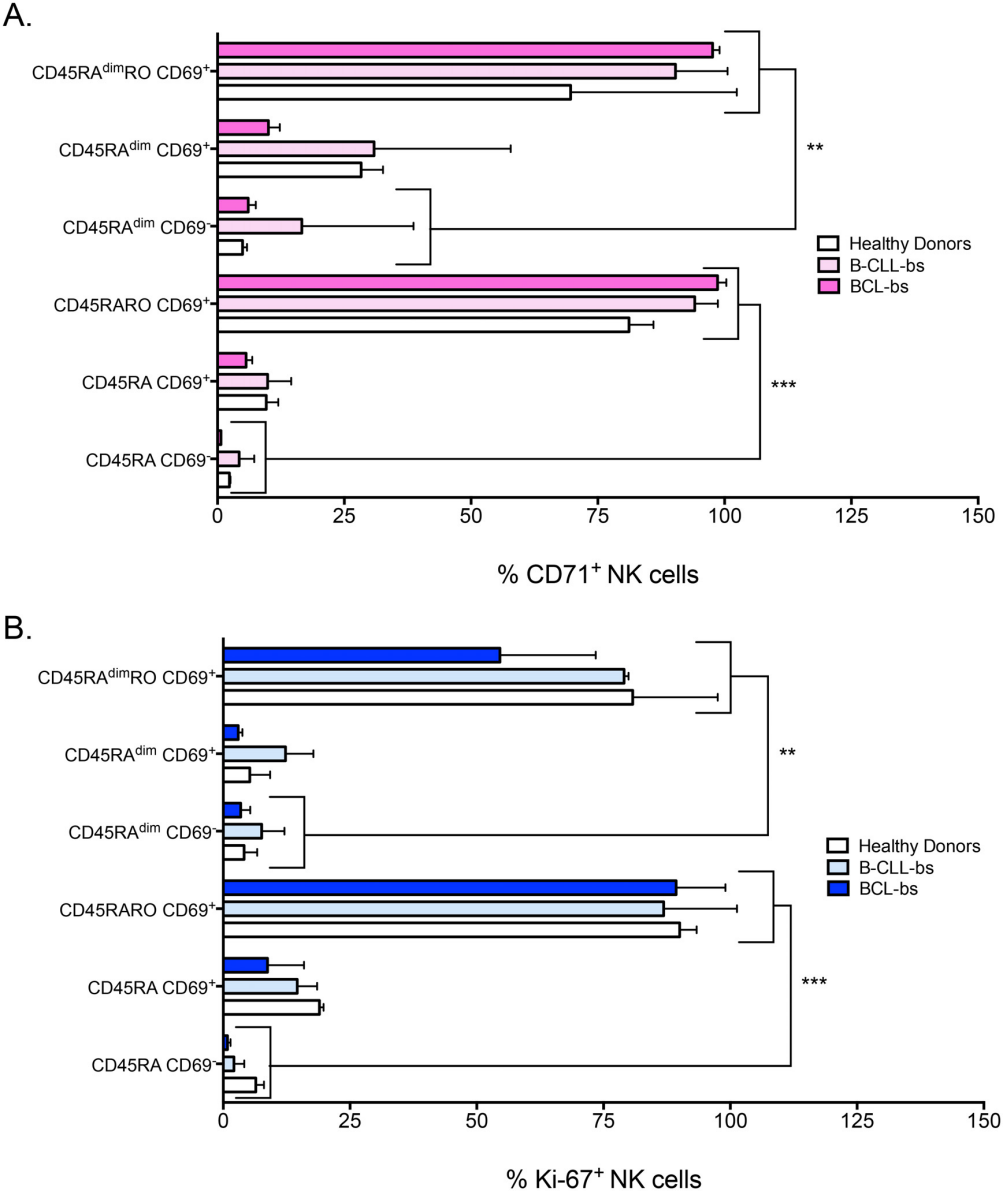
Lost of CD45RA or gain of CD69 does not correlate with metabolically active and proliferating NK cells. A-B) Percentage of NK cells that express CD71 or Ki-67 in the six different NK cell subsets (CD45/CD69) from blood samples of healthy donors (HD) and patients with B-cell chronic lymphocytic leukemia (B-CLL) or B-cell lymphoma (BCL). Bars represent the mean ± SD for each medical condition.

We then assessed the expression of transferrin receptor protein 1 (TfR1 or CD71), which is required for iron delivery from transferrin to the cells. CD71 expression increases in highly metabolic cells because iron is a cofactor for fundamental biochemical activities, such as oxygen transport, energy metabolism and DNA synthesis [34]. CD71 expression largely increased in cells expressing CD45RO, whereas CD69^+^ or CD45RA^dim^ cells in absence of CD45RO only marginally increased CD71 expression (Fig 6A). Similar results were obtained when the proliferation marker Ki-67 was analyzed (Fig 6B). This suggested that was CD45RO expression, and not the lost of CD45RA or the gain of CD69, which identified metabolically active, proliferating, NK cells in hematological cancer patients.

These results also suggested that CD45RA^dim^ cells could be an intermediate status for other subsets, but they are not a proliferating population generating other populations. CD45RA^dim^RO were probably CD45RARO cells that became exhausted after long interaction with target cells. In agreement with this idea, they express extremely low levels of GzmB although they have performed trogocytosis in targets cells and degranulated [17]. Moreover, they express low CD16 levels, a marker that is lost after prolonged NK cell activation [35, 36].

### 3.6. Several NK cell subsets from patients with hematological cancers show cytolytic activity against K562 cells *in vitro*

**Fig 7:**
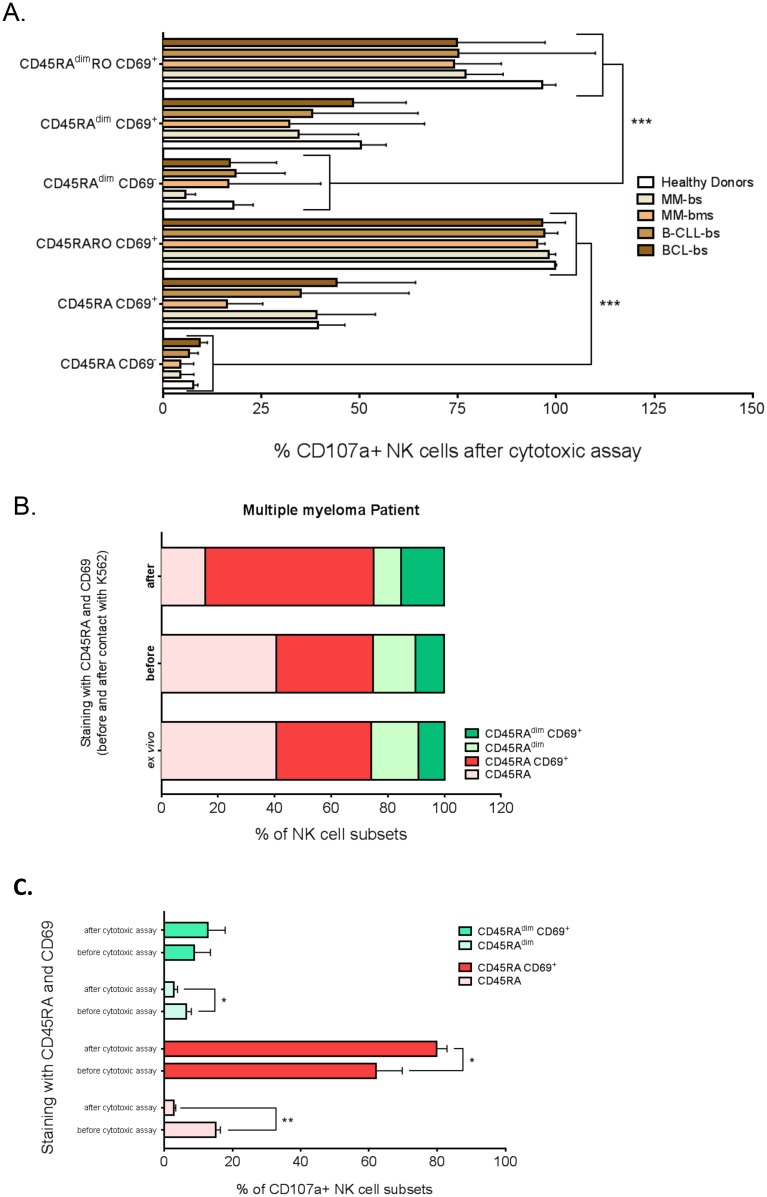
CD45RO^-^ and CD69^-^ cells can degranulate *in vitro*. PBMCs from healthy donors (HD) and patients with different hematological malignancies were purified as in Fig 1 and were incubated for 4 hours with target K562 tumor cells at the effector:target ratio of 10:1. A) Percentage of CD107a^+^ NK cells in the six different NK cell subsets (CD45RA/CD45RO/CD69 expression) described in Fig 5B. Bars represent the mean ± SD for each medical condition. B) Cells were treated as in A, but they were labeled with anti-CD45RA and–CD69 antibodies *ex vivo*, at the end of the *in vitro* cytotoxicity assay (after) or before the assay (before). The figure shows the percentage of cells in each NK cell subset in a patient with MM. C) Percentage of CD107a^+^ cells in each NK cell subset, based on antibody labeling before or after the *in vitro* cytotoxicity assay. The results represent the mean ± SD of 3 healthy donors.

To delineate which NK cell subsets from patients with hematological cancers have intrinsic degranulation activity, we incubated PBMCs from healthy donors or patients with K562 target cells (Fig 7A). These results were compared with the results *ex vivo* obtained on Fig 5B. The number of degranulating CD45RO cells, which was already very high *ex vivo* (Fig 5B), showed similar levels. The percentage of CD107a^+^ cells increased particularly in CD45RA^dim^ and CD69^+^ subsets (compare Figs 5B and 7A). Hence, all NK cell populations from patients with hematological malignancies partly retained the capacity to degranulate, remarkably CD45RA^dim^ cells.

Moreover, in our analysis we could have under-evaluated the percentage of CD69^-^ or CD45RA^dim^ cells that degranulated because after four hours of incubation with target cells the expression of CD69 and/or CD45 isoforms might have changed. We thus incubated PBMCs before with anti-CD69 and -CD45RA antibodies to determine the basal NK cell phenotype, and then we analyzed CD107a expression after exposure to K562 cells (Fig 7B and S3 Fig). Indeed, the NK cell profile before contact with target cells was similar to the phenotype observed *ex vivo*, showing that this was an efficient approach. After *in vitro* incubation with target cells (4 h), the number of CD69^+^ cells rapidly increased in both, the CD45RA and CD45RA^dim^ populations (about 100% increase in both subsets). However, the overall percentage of CD45RA and CD45RA^dim^ cells remained stable during this time (Fig 7B and S3 Fig). Hence, in contrast to CD69, CD45RA expression is not modified by activation at least in these settings. We realized that degranulation of CD69^-^ NK cells from healthy donors was higher than showed in Fig 7A (Fig 7C). In fact, 17% of CD45RACD69^-^ degranulated when we labeled cells before cytotoxic assay compared to 2% if we labeled later. Conversely, the opposite was found in CD45RACD69^+^ cells. Cells encountering the proper targets probably gained both CD69 and CD107a expression at the same time. This was also evident in NK cells from patients with hematological cancers in whom a large part of cells that degranulated were at the beginning CD69^-^ cells (S4 Fig). Something similar occurred in CD45RA^dim^ cells: more CD45RA^dim^CD69^-^ cells had degranulated than those observed when the labeling was a posteriori of the cytotoxic assay. The CD45RA^dim^ cells that gained CD69 expression after exposure to target cells were mainly CD56^dim^CD16^dim^ and fully mature CD56^dim^CD16^+^ (data not shown). In summary, CD45RA^dim^ cells can also respond to target cells at least by gaining CD69 and CD107a expression. Our results showed that degranulating cells acquired CD69. This would explain by *ex vivo* most CD107a^+^ cells were CD69^+^.

## Discussion

Identification of new human NK cell populations is important for understanding their physiology and for improving their therapeutic use in the clinic. We show here that like in T cells [27], CD45 increases in NK cells during maturation. Conversely, some CD45RA^dim^ cells, which show several features of immature cells, do not compensate CD45RA low levels with expression of CD45RO. These CD45RA^dim^ cells are also enriched in the CD56^bright^ compartment, which is usually linked to immature cells. It is believed that CD56^bright^ NK cells are precursors of CD56^dim^ NK cells, including *in vivo* [14]. This would suggest that these cells should proliferate and/or have an active metabolism. However, we show here that some of CD56^bright^ cells are quiescent. These CD45RA^dim^ cells can quickly react *in vitro* to target cells i.e. expression of CD69 and degranulation. However, *ex vivo* they do not show any of these features. We hypothesize that these dormant, CD45RA^dim^, cells can be a reservoir of NK cells without energetic cost for the host. These cells are largely expanded when the immune system is regenerating as in HSCT patients or under pathological conditions such as a hematological neoplasia.

Why are these cells quiescent? In view of the strong impaired function of CD45-deficient lymphocytes from human or mouse origin [20–24], it is tempting to speculate that a “minimal” level of the protein or of its activity is absolutely essential for lymphocyte function. The low CD45 expression in CD45RA^dim^ cells could be responsible of the quiescence of this population. It could be an economical way to have a population of cells ready to react but without consuming resources.

CD45 is required for full NK cell cytotoxicity *in vivo* in mice [37], but not *in vitro* [38–40]. In this context, CD45RA^dim^ cells show *in vitro* cytotoxicity; but *ex vivo* they do not show any sign of having performed *in vivo* cytotoxicity. This suggests that like mouse NK cells, human NK cells depend less on CD45 activity *in vitro* than *in vivo* to perform cytotoxicity. Hence, perhaps these cells will never show cytotoxic activity *in vivo*. If this is true, we would be confronted to inactivated or anergic NK cells that reach that status for an unknown reason.

Naive T lymphocytes usually express CD45RA and activated and memory T cells express CD45RO. Although it had been proposed that CD45RO expression identified memory NK cells [41], our previous [17] and current results strongly suggest that CD45RO in fact distinguished effector NK cells. Hence, could CD45RA^dim^ cells represent memory cells that will gain CD45RO after activation? We think that this is unlikely because they express mainly markers of immature cells. Hence, we propose that CD45RA^dim^ cells are a reservoir of NK cells without energetic cost for the host.

Finally, an important issue for the standard use of allogeneic NK cells in the clinic is the engraftment of an adequate number of cells that show clinically efficient anti-tumor activity. For this purpose, it is crucial to identify the different NK cell populations and their cytolytic activity. Here, we show that the expression of CD45 isoforms should facilitate this task and that, in principle, high amounts of CD45RA^dim^ cells are not the best option. However, it should be kept on mind that if these cells represent quiescent cells, they could be kept easier than other NK cell subsets and engrafted in patients where they could respond to target cells.

## Supporting Information

S1 Fig NK cell subsets in cancer patients allografted with HSC. A) After purification, PBMCs from allografted patients were stained as in Fig 2 and also with anti- CD62L, -CD57, -CD45RA, and -CD45RO antibodies.
(TIF)

S2 Fig Percentage of CD57^+^ and CD62L^+^ cells in different NK cell populations. Samples derived from blood (bs) or bone marrow (bms) samples from multiple myeloma patients.
(TIF)

S3 Fig CD69, but not CD45RA, expression changed during *in vitro* cytotoxicity. Cells were treated as in Fig 7 and labeled with anti-CD45RA and–CD69 antibodies *ex vivo*, at the end of the *in vitro* cytotoxicity assay (after) or before the assay (before). The figure shows the percentage of cells in each NK cell subset in two patients.
(TIF)

S4 Fig CD45RO^-^ and CD69^-^ cells can degranulate *in vitro*. Cells were treated as in Fig 7C. The figure shows the percentage of CD107a^+^ cells in each NK cell subset in three patients, based on antibody labeling before or after the *in vitro* cytotoxicity assay. The bars represent the mean ± SD.
(TIF)

S1 File Graphs and statistical analysis used in Fig 1D. (PZF)

S2 File Graphs and statistical analysis used in Fig 2A. (PZF)

S3 File Graphs and statistical analysis used in Fig 3. (PZF)

S4 File Graphs and statistical analysis used in Fig 4. (PZF)

S5 File Graphs and statistical analysis used in Fig 5. (PZF)

S6 File Graphs and statistical analysis used in Fig 6. (PZF)

S7 File Graphs and statistical analysis used in Fig 7. (PZF)

S1 Supporting Information Graphs and statistical analysis used in S2 Fig. (PZF)

S2 Supporting Information Graphs and statistical analysis used in S3 Fig. (PZF)

S3 Supporting Information Graphs and statistical analysis used in S4 Fig. (PZF)
