# Supplementary figures and images for: CD45 Isoform Profile Identifies Natural Killer (NK) Subsets with Differential Activity
Source: PLoS One. 2016 Apr 21;11(4):e0150434. doi: 10.1371/journal.pone.0150434 (PMC4839597; doi:10.1371/journal.pone.0150434)

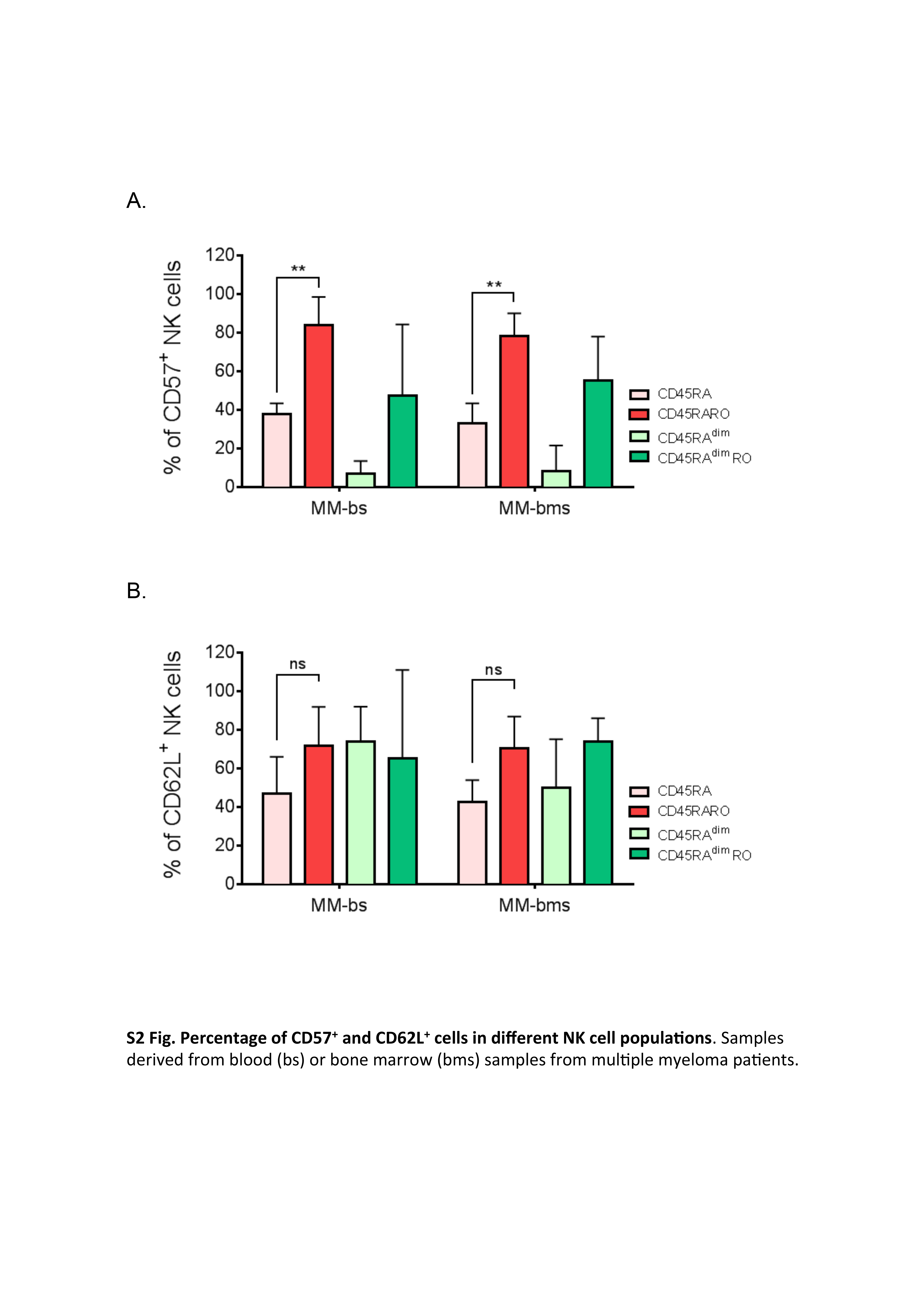

Supplement: S2 Fig — Samples derived from blood (bs) or bone marrow (bms) samples from multiple myeloma patients. (TIF) [file pone.0150434.s002.tif]

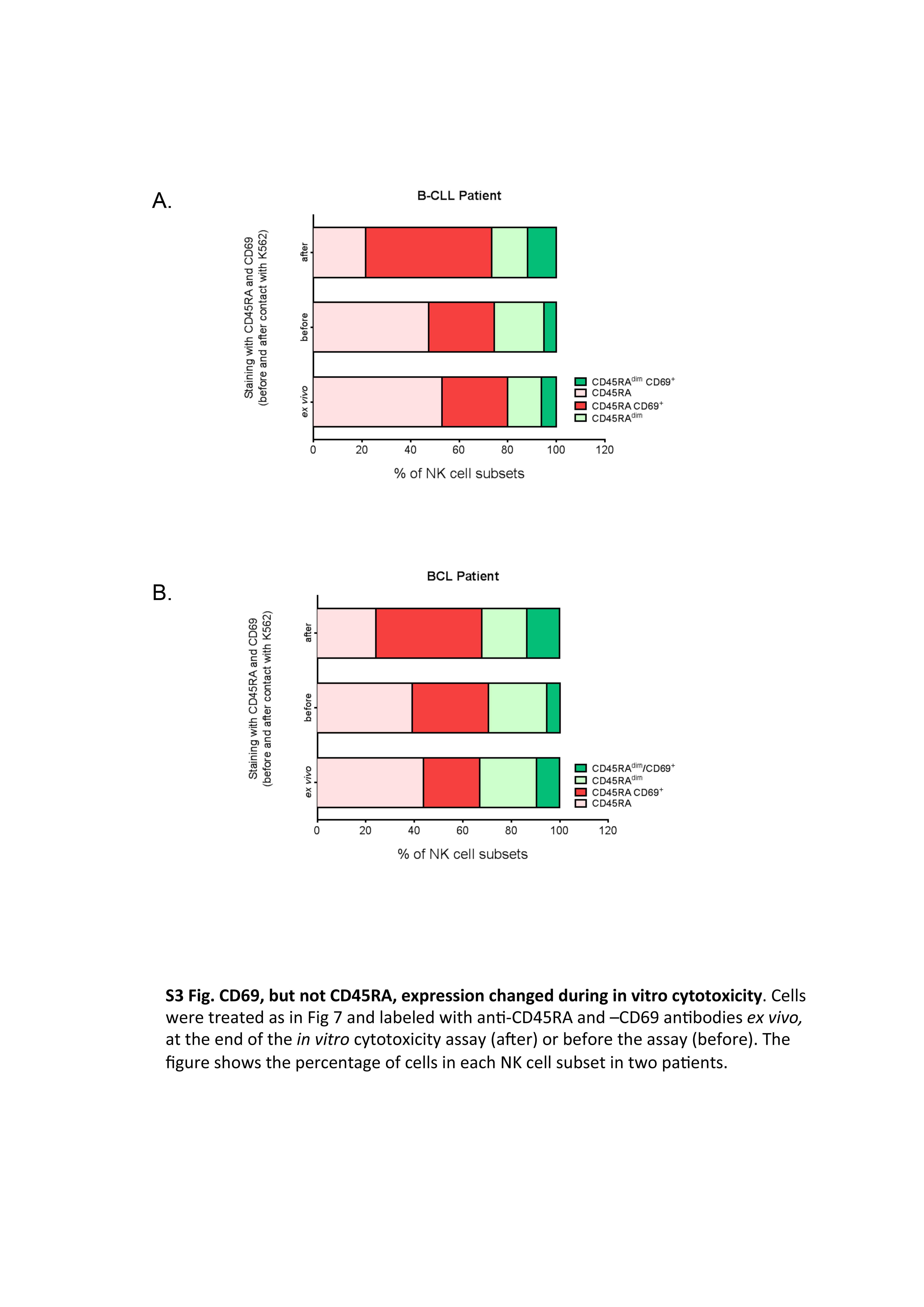

Supplement: S3 Fig — Cells were treated as in Fig 7 and labeled with anti-CD45RA and–CD69 antibodies ex vivo, at the end of the in vitro cytotoxicity assay (after) or before the assay (before). The figure shows the percentage of cells in each NK cell subset in two patients. (TIF) [file pone.0150434.s003.tif]

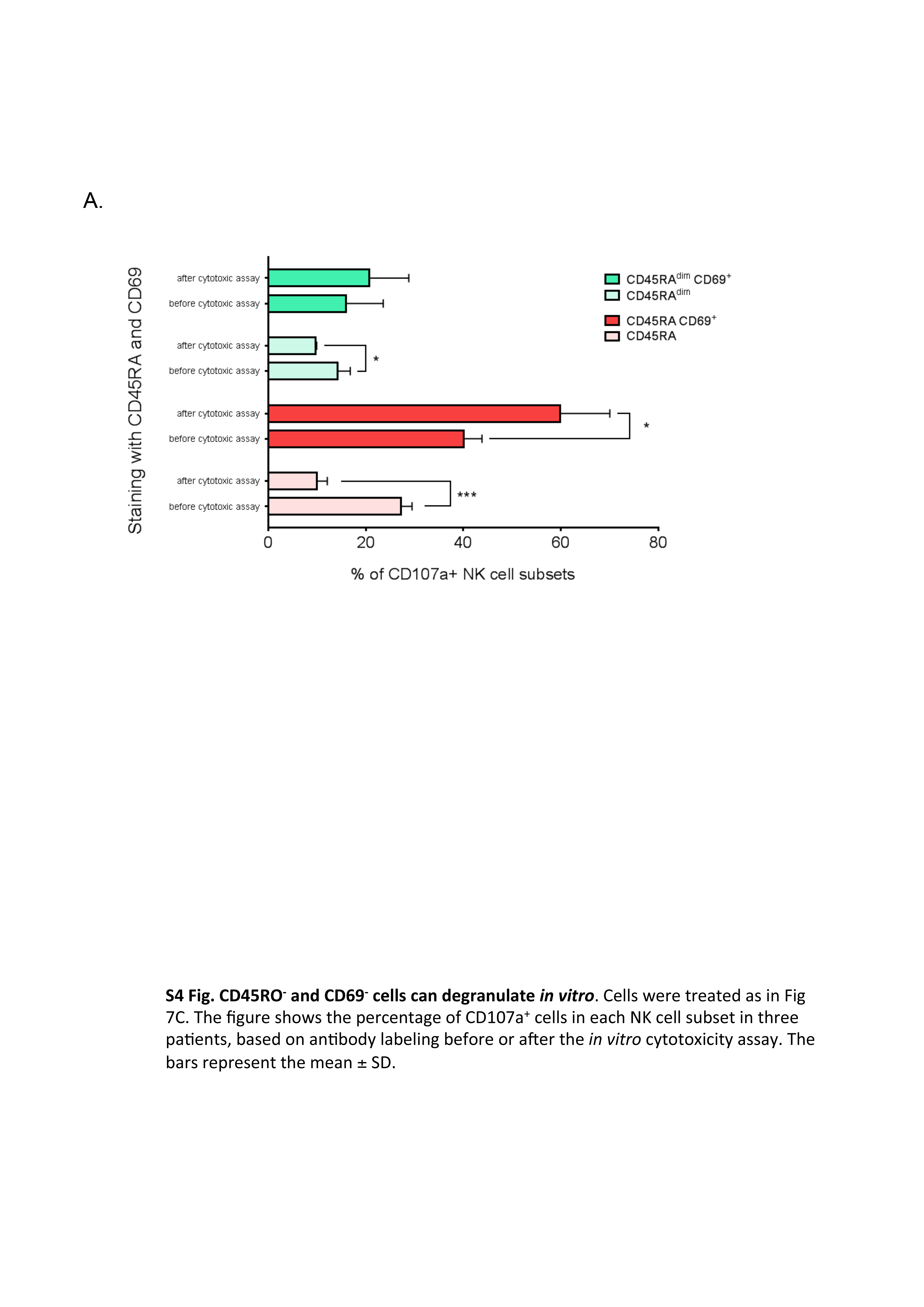

Supplement: S4 Fig — Cells were treated as in Fig 7C. The figure shows the percentage of CD107a+ cells in each NK cell subset in three patients, based on antibody labeling before or after the in vitro cytotoxicity assay. The bars represent the mean ± SD. (TIF) [file pone.0150434.s004.tif]
